# Quantum Dot‐Based Immunolabelling of Extracellular Vesicles and Detection Using Fluorescence‐Based Nanoparticle Tracking Analysis

**DOI:** 10.1002/jex2.70072

**Published:** 2025-07-22

**Authors:** Eunyong Ha, Yewon Han, Minseop Kim, Zayakhuu Gerelkhuu, Sook Jin Kwon, Tae Hyun Yoon

**Affiliations:** ^1^ Department of Chemistry, College of Natural Sciences Hanyang University Seoul Republic of Korea; ^2^ Research Institute for Convergence of Basic Science Hanyang University Seoul Republic of Korea; ^3^ Institute of Next Generation Material Design Hanyang University Seoul Republic of Korea; ^4^ Yoon Idea Lab. Co. Ltd Seoul Republic of Korea

**Keywords:** extracellular vesicles, fluorescence nanoparticle tracking analysis, immunolabelling, quantum dots

## Abstract

Extracellular vesicles (EVs) contain a variety of biomolecules, including DNA, RNA, lipids and proteins. They can interact with target cells to perform various functions, offering potential for therapeutic applications like drug delivery and diagnosis. The growing interest in EVs drives the need for robust methods for EV characterisation. One of the prevalent EV characterisation methods is scatter‐based nanoparticle tracking analysis (Sc‐NTA). This method measures the size and concentration of particles by tracking the scattered light from individual particles. However, Sc‐NTA has limitations in selectivity, as it detects all scattered light and fails to distinguish EVs from other nanoparticles, such as protein aggregates. To overcome this limitation, fluorescence‐based NTA (Fl‐NTA) is being utilised, where fluorescence tagging is used to selectively detect EVs. In previous studies, lipophilic dyes were employed for membrane labelling, but this resulted in false‐positive signals due to the staining of even non‐vesicular extracellular particles (NVEPs). Immunolabelling methods using antibodies that specifically bind to EV‐specific protein were also introduced; yet challenges with sensitivity and photostability of the organic dyes remained. To address the challenges, we conjugated quantum dots (QDs) to antibodies that specifically bind to EV‐specific markers, CD9, CD63 and then immunolabelled the EVs. Labelling conditions were optimised to develop a robust protocol for QD‐based immunolabelling. Detection sensitivity was evaluated by comparing QD‐based immunolabelling with Alexa dye‐based methods. Furthermore, size distribution analysis demonstrated the ability of QDs to detect smaller EV populations. Finally, subpopulations of EVs from various cell lines were profiled. This approach enhances the accurate characterisation of EVs, providing a reliable and reproducible method for EV quality control and improved insights into their heterogeneity.

## 1 Introduction

Cells release extracellular vesicles (EVs), which are phospholipid bilayer‐structured particles, into their surrounding environment (van Niel et al. 2018). EVs carry a distinct set of lipids, nucleic acids and proteins derived from their cell of origin. Once released, EVs interact with specific cells, transferring a complex array of molecular information and carrying out various biological functions (Kalluri and LeBleu 2020). Given their critical role in intercellular communication, EVs hold significant potential as innovative biomarkers for diagnosis and drug delivery carriers (Luo et al. 2021; Nam et al. 2020; Boukouris and Mathivanan 2015). However, the significant heterogeneity of EVs poses challenges to their application in these areas. This heterogeneity is evident both in their size (‘operational terms’) and their mode of biogenesis (e.g., exosomes, ectosomes and apoptotic bodies—‘biogenetic terms’) (Welsh et al. 2024).

Given their heterogeneity, the need for precise, reliable and accurate methods to determine their size, concentration and subpopulation has become essential (van de Wakker et al. 2023). Scatter‐based nanoparticle tracking analysis (Sc‐NTA) is one of the most common methods for characterising EVs. It works by tracking the Brownian motion of individual particles, allowing for size determinations based on the Stokes‐Einstein equation (E56 Committee n.d.; Dragovic et al. 2011). Additionally, the camera records the number of particles per frame over a set period, estimates the particle concentration within a unit volume. Sc‐NTA offers the advantage of real‐time, single‐EV‐level particle tracking, providing quantitative data on particle size and concentration. However, its inability to distinguish EVs from similarly sized non‐vesicular particles limits its selectivity. Sc‐NTA detects scattered light from all particles, not just EVs, making it challenging to selectively detect only EVs. To overcome this limitation, fluorescence based NTA (Fl‐NTA) is being utilised, where fluorescence tagging combined with a long‐pass filter enables the selective detection of labelled EVs (Carnell‐Morris et al. 2017). This approach allows fluorescently tagged EVs to be distinguished from co‐isolated particles such as lipoproteins and protein contaminants, thereby enhancing the specificity and reliability of EV analysis.

However, conventional methods for labelling EVs and Fl‐NTA analysis still face several challenges and limitations (Gangadaran et al. 2017). Common lipophilic dyes (e.g., R18, DiI, PKH26, PKH67 and CellMask) label EV, lipid membranes, but are also prone to staining non‐vesicular extracellular particles (NVEPs), such as exomeres and supermeres. These NVEPs, recently identified as single‐layer membrane particles overlapping in size with EVs, can result in false‐positive signals (Welsh et al. 2024; Takov et al. 2017; Zhang et al. 2023). To overcome these issues, labelling methods using antibodies targeting EV‐specific proteins, such as the tetraspanins CD9, CD63 and CD81, have been utilised (Mondal et al. 2019). However, the organic dyes used in this approach often lack sufficient brightness and are prone to photobleaching (Montón et al. 2009) under extended laser exposure in NTA, leading to signal loss during prolonged measurements.

Given these limitations, quantum dots (QDs) present a promising alternative for the labelling and tracking of EVs. These semiconductor nanocrystals provide unique optical properties, including high brightness and exceptional photostability (Montón et al. 2009), making them highly suitable for precise and long‐term tracking. Importantly, QDs enable effective detection of EVs within a size range of approximately 30–200 nm and provide a broad linear dynamic range for accurate particle quantification. Previous studies have demonstrated the utility of QD‐conjugated antibodies in combination with NTA for EV analysis, laying the groundwork for their application in profiling EV subpopulations (Oosthuyzen et al. 2013; Gercel‐Taylor et al. 2012; McNicholas and Michael 2017). However, the workflow for immunolabelling using QDs, robust Fl‐NTA measurement and subsequent data analysis has not yet been established. Furthermore, comparative analyses of EV subpopulations using QD conjugated antibodies have not been widely conducted.

In this study, we optimised the immunolabelling protocols using QD conjugated antibodies and Fl‐NTA analysis. We also aimed to evaluate how the use of QDs as fluorophores improves detection sensitivity, specifically in terms of number concentration (detectable particles) and *size (lower size range)* (International Organization for Standardization 2016). To achieve this, we compared the number concentration of EVs immunolabelled with different fluorophores (QD625 vs. Alexa 488) and analysed the size distribution using different NTA modes (Scatter vs. Fluorescence). Furthermore, using this method, we compared EV subpopulations from various cell lines (THP‐1 vs. EA.hy926 vs. A549). This improved approach enables more reliable and reproducible EV characterisation, contributing to enhanced EV quality control and better understanding of EV heterogeneity.

## 2 Materials and Methods

### 2.1 Cell Culture and EV Enrichment

A549, THP‐1 and EA.hy926 cell lines were obtained from the American Type Culture Collection (ATCC). A549 and THP‐1 cells were cultured in complete media consisting of 89% Roswell Park Memorial Institute (RPMI)‐1640, 10% foetal bovine serum (FBS, Ref#: 16000–044, Gibco) and 1% antibiotic‐antimycotic. EA.hy926 cells were cultured in Dulbecco's Modified Eagle Medium (DMEM) under the same conditions. All cells were cultured under standard conditions at 37°C with 5% CO_2_. When cell confluency reached ∼80%, the cells were washed three times with Dulbecco's phosphate‐buffered saline (DPBS, Cat#: LB 001–02, Welgene) to remove FBS‐derived EVs. Afterward, the medium was replaced with one containing Exosome‐Depleted FBS (Ref#: A27208‐03, Gibco) and the cells were incubated for an additional 24 h as a part of the EV enrichment process. To confirm the absence of contaminating particles, Exosome‐depleted FBS was analysed by NTA prior to use. The resulting particle distribution is shown in **Figure** **S6**, confirming minimal background signal.

These cell lines were selected to represent lung epithelial (A549), monocytic (THP‐1) and endothelial (EA.hy926) components of the pulmonary microenvironment, reflecting the structure of a 3D lung model in our laboratory.

### 2.2 EV Isolation

EV isolation was performed using a polyethylene glycol (PEG)‐based precipitation method (Total Exosome Isolation, Cat#: 4478359, Invitrogen). The detailed workflow followed the manufacturer's protocol. In brief, EV enriched media from adherent cells (A549, EA.hy926) and suspension cells (THP‐1) were centrifuged at 800 × *g* for 5 min to remove cells, followed by centrifugation at 2000 × *g* for 30 min to remove cell debris. Supernatant was carefully taken and then mixed with PEG reagent at a 2:1 ratio and then incubated at 4°C overnight. After incubation, the samples were centrifuged at 10,000 × *g* for 1 h at 4°C. The supernatant was aspirated, leaving the EVs in the pellet at the bottom of the tube. The pellet was resuspended in sterile‐filtered DPBS for subsequent experiments.

### 2.3 Quantum Dot Conjugation to Antibodies

Monoclonal mouse anti‐human CD63 antibody (IgG1, clone#: 460305, Cat#: MAB5048, R&D Systems) and monoclonal mouse anti‐human CD9 antibody (IgG2B, clone#: 209306, Cat#: MAB1880‐100, R&D Systems) were used for immunolabelling. Antibodies were conjugated to QD625 nanocrystals (SiteClick Antibody Labelling Kit, Cat#: S10452, Thermo Fisher Scientific). Briefly, the process of antibody conjugation was carried out using SiteClick coupling procedure, which involved the attachment of a dibenzocyclooctyne (DIBO) modified QD625 with an azide modified anti‐CD9 and anti‐CD63 antibodies. The procedure followed the standardised manufacturer's protocol. The core size of QDs was determined using TEM (JEM 2100F; JEOL), as shown in Figure S1. The hydrodynamic size of QD‐conjugated antibodies was measured using dynamic light scattering (DLS, Zetasizer Nano, ZS90; Malvern Panalytical). The concentration of the QD conjugated antibodies was measured using UV/Vis spectrometer (NanoDrop One; Thermo Fisher Scientific). To compare between QD and organic dyes, Alexa Fluor 488‐conjugated monoclonal mouse anti‐human CD63 antibody (IgG1, clone#: H5C6, Cat#: 353037, BioLegend) and Alexa Fluor 488‐conjugated monoclonal mouse anti‐human CD9 antibody (IgG2B, clone#: 209306, Cat#: FAB1880G, R&D Systems) were also used.

### 2.4 EV Immunolabelling

The concentration of unlabelled EVs was first measured using NTA, and the samples were diluted to 2 × 10⁹ particles for labelling. This particle number was chosen to ensure that sufficient EVs would remain after the subsequent washing steps for accurate Fl‐NTA analysis. To optimise the immunolabelling conditions, experiments were conducted by varying the concentration of QD conjugated antibodies (0.01, 0.1, 0.5, 1 and 2 µL), staining volumes (20, 100 and 1000 µL) and staining times (1, 6, 12 and 24 h). The antibody concentrations were 0.90 ± 0.02 µM for anti‐CD9 and 1.90 ± 0.20 µM for anti‐CD63, as determined by Nanodrop spectrophotometer (Thermo Fisher Scientific). Following staining, unbound antibody‐QDs were removed using PEG precipitation: labelled EV were diluted in PBS (600–1000 µL), mixed with PEG at 1:2 (PEG:sample) volume ratio, and incubated at 4°C for 24 h. The EVs were pelleted at 10,000 × *g* and resuspended in 1 mL of DPBS for analysis.

### 2.5 NTA Setting

All particle tracking analyses were performed using a NTA (NS300; Malvern Panalytical) equipped with long‐pass filter. EV concentrations were measured using sc‐NTA, and the samples were diluted to between 1 × 10^8^ and 1 × 10^9^ particles/mL, based on instrument guidelines. In the fluorescence mode, a 430 nm long‐pass filter was used for QD625 (excitation: 405 nm; emission: 625 nm), and a 500 nm long‐pass filter was used for Alexa 488 (excitation: 488 nm, emission: 520 nm). A syringe pump was used in both modes, at a flow rate of 50. Each analysis was carried out in triplicate, with 90 s videos. Each video required a minimum of 500 valid particle tracks, yielding at least 1500 tracks per sample. Data were processed using NanoSight software (version 3.4) with default settings. The camera levels were manually fine‐tuned between 15 and 16 to enhance the visibility of particles by optimising light scattering. The detection threshold was set to 4–5 to increase sensitivity while minimising background noise. To ensure consistency, these settings were kept constant across samples from the same source. The accuracy of particle size measurement by the NS300 system was validated using 50 and 100 nm Fluoresbrite Yellow Green polystyrene beads (Cat# 17149‐10 and 17150‐10, respectively; PolyScience) (**Figure** **S2**).

### 2.6 EV Characterisation Using Western Blot and SEM

EV proteins from A549 cells were extracted using RIPA buffer (Rockland, Lot#: 49818), denatured at 98°C for 5 min, and loaded at 30 µg per well. The proteins were separated by 4%–10% SDS‐PAGE and transferred to membranes (20 min at 60 mA, followed by 60 min at 100 mA). The membranes were then blocked with 5% skim milk and 0.05% Tween‐20 in tris buffered saline (TBS, BioRad) for 1 h at room temperature, and incubated overnight at 4°C with rabbit primary antibodies (CD9, CD63, HSP70, Cat#: ab275018, Abcam), diluted to 1:1000. After washing with 0.01% Tween‐20 in TBS, the membranes were exposed to secondary antibodies conjugated to horseradish peroxidase diluted to 1:10,000 for 1 h at room temperature. The reaction products were visualised using a ChemiDoc (Fusion SL, Viber Lourmat). For scanning electron microscope (SEM) analysis, 1 × 10^9^ EVs were deposited onto silicon wafers, dried for 24 h, and sputter‐coated with gold for 3 min. SEM imaging was conducted using a Carl Zeiss Sigma 300 equipped with an EDS system (AZtecLive Lite/Ultim Max 40), using: 3.00 kV accelerating voltage, 4.5 mm working distance, and InLens detector mode.

### 2.7 Statistical Analysis

NTA results are shown as mean ± standard error (SE) to reflect the precision of the average from replicate measurements, while all other data are presented as mean ± standard deviation (SD). Statistical significance was determined using an unpaired *t*‐test with GraphPad Prism Version 10.3.1. Statistical significance is indicated by **** (*p* < 0.0001) and *** (*p* < 0.001).

## 3 Results and Discussion

**FIGURE 1:**
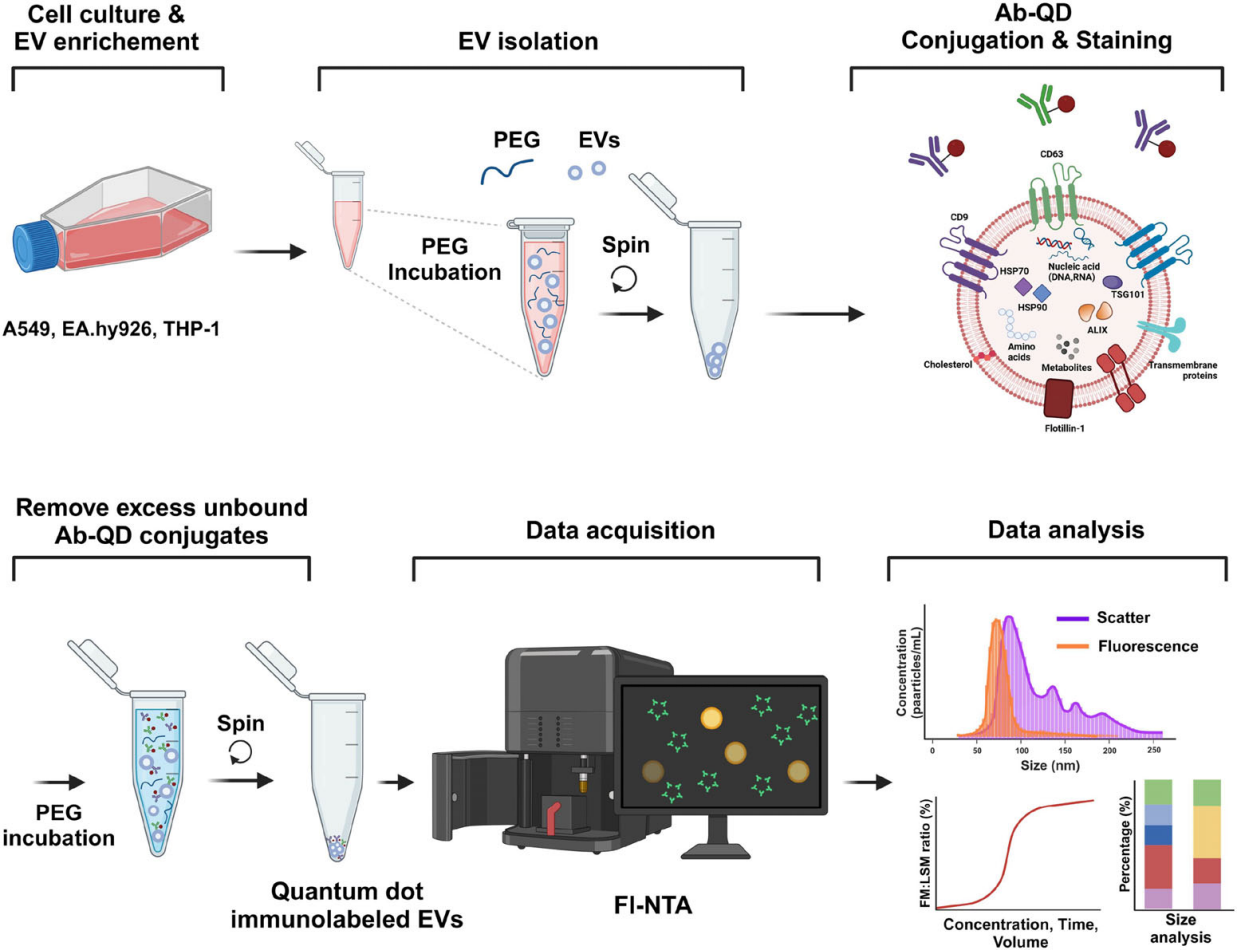
Overall workflow of EV immunolabelling and Fl‐NTA analysis. Cells are cultured, and EVs are enriched from the cell culture medium. Then polyethylene glycol (PEG) is added to precipitate EVs, followed by centrifugation to isolate EVs. Quantum dots (QDs) are conjugated to anti‐CD9 and anti‐CD63 antibodies for specific labelling of EV surface markers. EVs are immunolabelled with QD conjugated antibodies, excess dyes are removed through additional washing steps. Finally, labelled EVs are resuspended for further analysis. Labelled EVs are analysed using Sc‐/Fl‐NTA. The analysis includes optimisation of labelling conditions, size analysis and comparative studies on different fluorophores, EV markers and cell lines.

**Figure** **1** provides an overview of the experimental workflow used in this study. It illustrates the stepwise process of EV enrichment from three different cell lines, PEG‐based EV isolation, quantum dot (QD)‐conjugated antibody immunolabelling, fluorescence‐based nanoparticle tracking analysis (Fl‐NTA), and subsequent comparative analysis across cell types. This schematic outlines the core methodology optimised and applied throughout the study.

### 3.1 Characterisation of EVs

**FIGURE 2:**
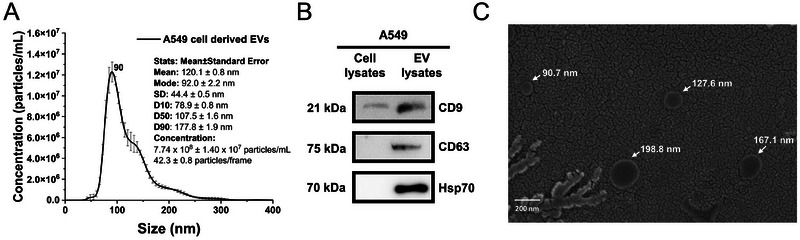
Characterisation of EVs derived from A549 cells. (A) Sc‐NTA indicating the size distribution of A549‐derived EVs, with the majority falling within the 30–200 nm range. (B) Western blot demonstrating the enrichment of EV‐specific markers, including CD9, CD63 and Hsp70, in EVs isolated from A549 cells, thereby confirming the successful isolation of EVs. (C) SEM image showing the morphology and size of EVs derived from A549 cells, revealing their characteristic spherical shape and nanoscale size.

EVs derived from A549 cells were isolated using PEG precipitation and characterised through NTA, Western blot and SEM. NTA was used to measure size distribution and determine number concentration of A549 cell derived EVs. As shown in **Figure** **2A**, the NTA provided detailed metrics, including the mean, mode, standard deviation, D10, D50, D90 size and particle concentration. The results revealed that 93.7% of the detectable particles in scatter mode fell within the size range of approximately 30–200 nm, which corresponds to the typical size range of small EVs (Luo et al. 2021; Möller and Lobb 2020). Western blot analysis was performed to compare A549 cell lysates with EV lysates. As shown in **Figure** **2B**, the results demonstrated that EV‐specific proteins, including CD9 (21 kDa), CD63 (75 kDa) and Hsp70 (70 kDa), were significantly enriched in the EV lysates compared to the cell lysates. This indicates that the majority of the isolated proteins are associated with EVs, validating the isolation of EVs. The SEM image confirmed the presence of particles with structures within the size range typical of EVs, consistent with the NTA results (**Figure** **2C**).

To further evaluate the purity of the isolated A549‐derived EVs, we performed a Bradford assay to quantify total protein content. The results, presented in **Figure** **S4**, showed a protein level comparable to that typically observed with ultracentrifugation‐based EV isolation (Webber and Clayton 2013). Nonetheless, we acknowledge that PEG‐based precipitation may co‐isolate non‐EV protein contaminants, and does not completely exclude soluble protein complexes or lipoproteins. While suitable for downstream labelling optimisation, this method may be less appropriate for applications requiring highly pure EVs. Alternative orthogonal methods such as tangential flow filtration (TFF) or size exclusion chromatography (SEC) could offer improved EV purity and are recommended for future studies aimed at functional characterisation.

### 3.2 Optimisation of EV Immunolabelling Conditions

After performing EV isolation and characterisation, we optimised conditions for EV immunolabelling to enhance the reproducibility of our protocol. While the high sensitivity of the NTA in fluorescence mode (FM) is high enough to detect fluorescence signal from QDs, on the other hand it also means a higher susceptibility to background noise from QDs that were not completely removed during the washing process (Dlugolecka and Czystowska‐Kuzmicz 2024). In particular, unwashed QDs can significantly influence the size distribution, particle concentration and size of detected particles (Welsh et al. 2024). This underscores the critical importance of implementing thorough washing steps during the immunolabelling process. Size exclusion chromatography and tangential flow filtration are commonly used size‐based methods for separating EVs and QDs (Zhang et al. 2020; Midekessa et al. 2021). However, these approaches can result in the loss of small EVs depending on the size cutoff. To address this, we evaluated the use of PEG precipitation method, confirming that the washing process effectively removed the QDs.

**FIGURE 3:**
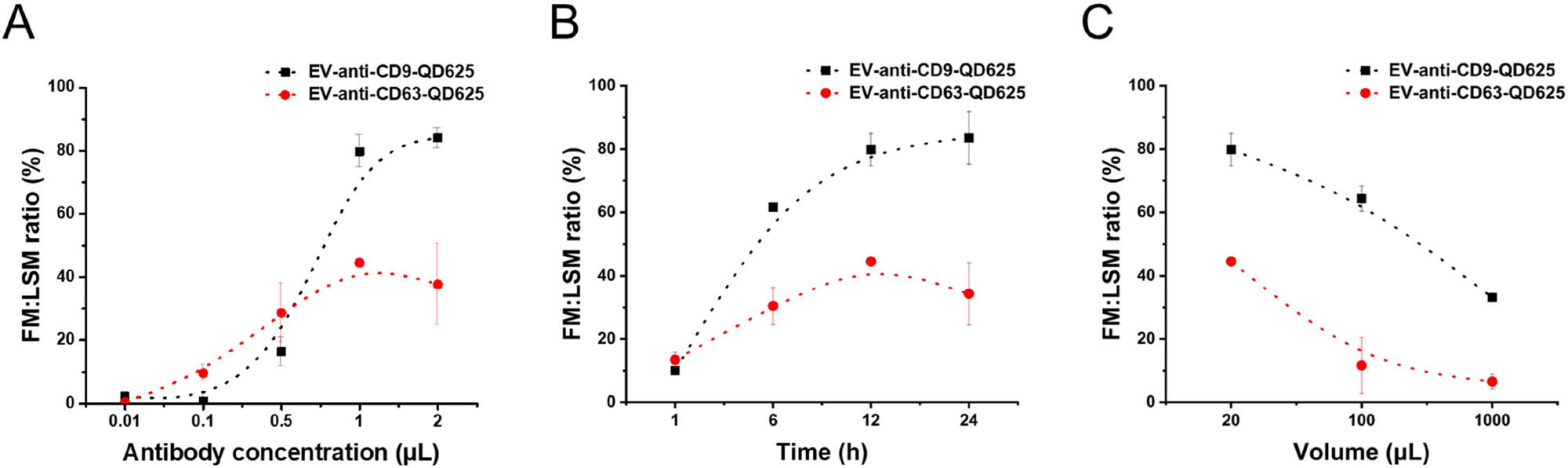
Optimisation of EV immunolabelling conditions. Evaluated by varying (A) antibody dilution, (B) incubation time and (C) staining volumes. According to Nanodrop measurements, the concentrations of the antibodies were as follows: for anti‐CD9 antibody corresponds to 0.90 ± 0.02 µM, and for anti‐CD63 antibody corresponds to 1.90 ± 0.20 µM. Immunolabelling efficiency is represented as the ratio of the number concentration in fluorescence mode to that in light scatter mode, serving as an indicator of labelling success. The lines represent B‐spline interpolation, applied to the experimental data points to visualise the overall trend in labeling efficiency. Error bars represent SD (*n* = 3).

Given the small size of EVs, careful optimisation is required, as their labelling differs significantly from standard cell labelling protocols (Welsh et al. 2024). Optimisation of antibody concentration is crucial not only to ensure reproducibility, obtain optimal signals and prevent antibody wastage. Additionally, structural differences and Fc receptor binding characteristics of each antibody can influence specificity and background noise (Mondal et al. 2019). To address this, we optimised the concentration of anti‐CD9‐QD625 and anti‐CD63‐QD625 conjugates by varying staining conditions (González‐Domínguez et al. 2020). Staining was performed to target the surface proteins CD9 and CD63 on EVs. After staining, excess antibody conjugates were removed using the previously established washing protocol. EVs immunolabelled with QDs were measured using Fl‐NTA in both fluorescence and scatter modes, and the immunolabelling efficiency was calculated as the ratio of number concentration of fluorescence to scatter mode (Thane et al. 2019). Antibody concentrations were set at 0.01, 0.1, 0.5, 1 and 2 µL. As shown in **Figure** **3A**, the maximum immunolabelling efficiency was achieved at 1 µL for both antibodies. Additionally, the immunolabelling efficiency for CD9 and CD63 differed significantly, with efficiencies of 79.8% and 44.5%, respectively. This suggests that CD9‐positive EVs are more abundant than CD63‐positive EVs in A549‐derived EVs. To further evaluate the effect of staining time, experiments were conducted under different incubation times: 1, 6, 12 and 24 h. As shown in **Figure** **3B**, it was determined that a minimum staining time of 12 h is required to achieve the highest immunolabelling efficiency. Finally, to evaluate the effect of staining volume, staining was performed with 20, 100 and 1000 µL. **Figure** **3C** showed that the highest immunolabelling efficiency was achieved at a reaction volume of 20 µL. This indicates that the staining volume significantly impacts labelling efficiency, likely due to its influence on the interaction dynamics between EVs and antibodies. These optimised conditions establish our protocol as a robust and reproducible method for EV immunolabelling, which can be applied in future studies to improve consistency and reliability. Based on these optimised conditions, we proceeded with further analyses.

### 3.3 Improvement in Detection Sensitivity of Fl‐NTA Using Quantum Dots

After optimising EV immunolabelling with QD conjugated antibodies, we investigated how the limitations of organic fluorescent dyes—such as reduced brightness and poor photostability, as identified in previous studies—affect NTA measurements. Furthermore, we sought to explore how QDs differ from these traditional fluorophores in addressing these challenges. These limitations are particularly critical in immunolabelling, where the number of CD9 and CD63 antigens expressed on EVs is a key factor (Mathieu et al. 2021; Ekström‐ 2022 ‐ Characterization of surface markers on extracellul.pdf Koch et al. 2021). Unlike membrane labelling, which stains the entire EV membrane, immunolabelling targets specific antigens like CD9 and CD63, which are typically present in low abundance on EVs (Nolan and Jones 2017). As a result, fewer fluorophores can bind, and an insufficient fluorescent signal may hinder particle detection, reducing sensitivity and accuracy. Therefore, achieving a strong fluorescence signal is critical for reliable immunolabelling results.

**FIGURE 4:**
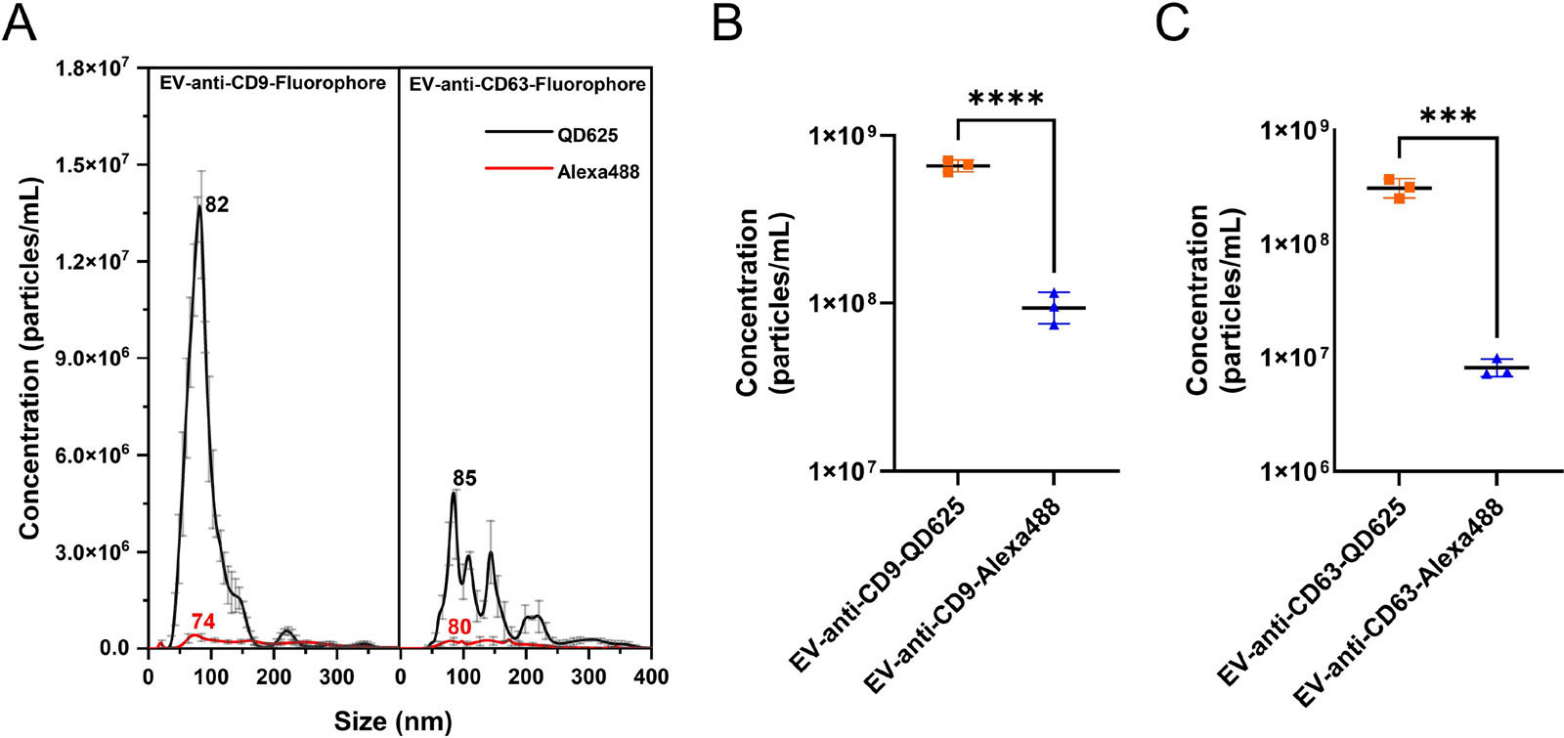
Size distribution of A549 cell‐derived EVs immunolabelled with QD625 and Alexa488. (A) The size distribution of EVs were measured using Fl‐NTA to compare the performance of QD625 (black line) and Alexa488 (red line) as fluorescent labels. QD625‐labelled EVs exhibited significantly higher number concentration compared to Alexa488‐labelled EVs, reflecting QD's superior fluorescence intensity and suitability for Fl‐NTA measurements. Error bars represent SE (*n* = 3). The mode sizes of labelled EVs are annotated in the plot. (B, C) showing significant difference in number concentration between EVs immunolabelled with QD625 and Alexa488. Statistical significance is denoted by **** (*p* < 0.0001) and *** (*p* < 0.001).

To address these challenges and evaluate the suitability of QDs as an alternative to conventional fluorescent dyes, we compared the number concentration of EVs stained with anti‐CD9‐QD625 and anti‐CD63‐QD625 versus those stained with anti‐CD9‐Alexa488 and anti‐CD63‐Alexa488 using Fl‐NTA. As shown in **Figure** **4A**, the size distributions of CD9‐positive and CD63‐positive EVs stained with different fluorescent dyes are presented. Figure 4B,C further highlight the significant differences observed. In both cases, a significantly higher number of EVs were detected when QD625 was used as the fluorescent label compared to Alexa488. Although Alexa488 is widely utilised in various analytical techniques, such as confocal microscopy (Bağcı et al. 2022) and flow cytometry (Mladenović et al. 2025), its application in Fl‐NTA is constrained by insufficient brightness to meet the sensitivity requirements of the detector. Additionally, tracking particles across multiple frames demands high photostability, further emphasising the limitations of Alexa488 for Fl‐NTA. As shown in **Figure** **S5**, overlay analysis of fluorescence and scatter signals for QD625‐labelled EVs confirms a strong co‐localisation, reinforcing the specificity and stability of the immunolabelling approach. In contrast, QDs are already known to outperform Alexa dyes in terms of both brightness and photostability under prolonged laser exposure (Montón et al. 2009). As demonstrated by our results, QDs proved to be more suitable for Fl‐NTA measurements.

As shown in **Table** **S1**, the ratio of particle concentrations in FM to LSM was significantly lower when Alexa dyes were used—approximately 11.5% for anti‐CD9‐Alexa488 and 5.9% for anti‐CD63‐Alexa488, whereas QDs achieved substantially higher ratios. Moreover, when Alexa488 was used, the number of detected EVs in FM with valid statistical tracks was fewer than 200, further indicating lower sensitivity. In contrast, QD625 labelling enabled the detection of a significantly higher number of particles with robust statistical reliability. These findings underscore the superior fluorescence signal and sensitivity of QD625, establishing it as a more reliable label for Fl‐NTA.

While the size distribution will be discussed in detail in the next section, we briefly compared the size changes following QD and Alexa immunolabelling. As shown in **Table** **1**, the mean and mode sizes in scatter mode indicate that QD‐labelled EVs increased in size compared to unstained EVs, whereas Alexa‐labelled EVs showed minimal changes in size. Since the size distribution differs from that of unlabelled EVs, careful size analysis should be accompanied.

**TABLE 1:** Comparison of particle sizes of A549 cell‐derived EVs immunolabelled with QD625 and Alexa488.

NTA measurement mode	Size	A549 Cell‐derived EVs				
		Unstained	QD625 labelled		Alexa488 labelled	
			CD9‐(+)	CD63‐(+)	CD9‐(+)	CD63‐(+)
Scatter mode	Mean (nm)	120.1 ± 0.8	141.4 ± 1.1	156.0 ± 2.7	120.7 ± 1.3	126.9 ± 3.6
	Mode (nm)	92.0 ± 2.2	90.3 ± 1.4	118.1 ± 6.0	88.1 ± 5.5	80.8 ± 5.4
Fluorescence mode	Mean (nm)	—	95.7 ± 3.1	141.9 ± 2.7	207.2 ± 4.2^a^	293.0 ± 75.2^a^
	Mode (nm)	—	77.9 ± 3.1	103.8 ± 19.7	92.2 ± 19.8^a^	129.1 ± 26.9^a^

^a^ The valid track counts are below 200.

### 3.4 Comparison of Size Distribution of QD Immunolabelled EV in FM and LSM

Thus far, we examined the improvement in detection sensitivity in terms of particle concentration. In this section, we shift our attention to size sensitivity, specifically the improvement in detecting smaller EV sizes. The key considerations during size analysis include comparing LSM and FM with particular attention to the influence of QDs. As shown in **Figure** **S1**, the core size of QDs was determined to be 8.5 ± 1.3 nm using TEM. After conjugation with antibodies, their hydrodynamic size was measured using NTA as 14.8 ± 1.0 nm for anti‐CD9‐QD625 conjugates and 13.3 ± 1.2 nm for anti‐CD63‐QD625 conjugates. The size measured by DLS for anti‐CD9‐QD625 was 19.2 ± 8.1 nm. These size differences may influence the EV size when QDs are attached to EVs. To account for these effects, we conducted three comparisons: NTA measurement of (1) unlabelled EVs (in LSM) versus labelled EVs (in FM), (2) labelled EVs (in LSM) versus labelled EVs (in FM) and (3) labelled EVs (in LSM) versus unlabelled EVs (in LSM).

**FIGURE 5:**
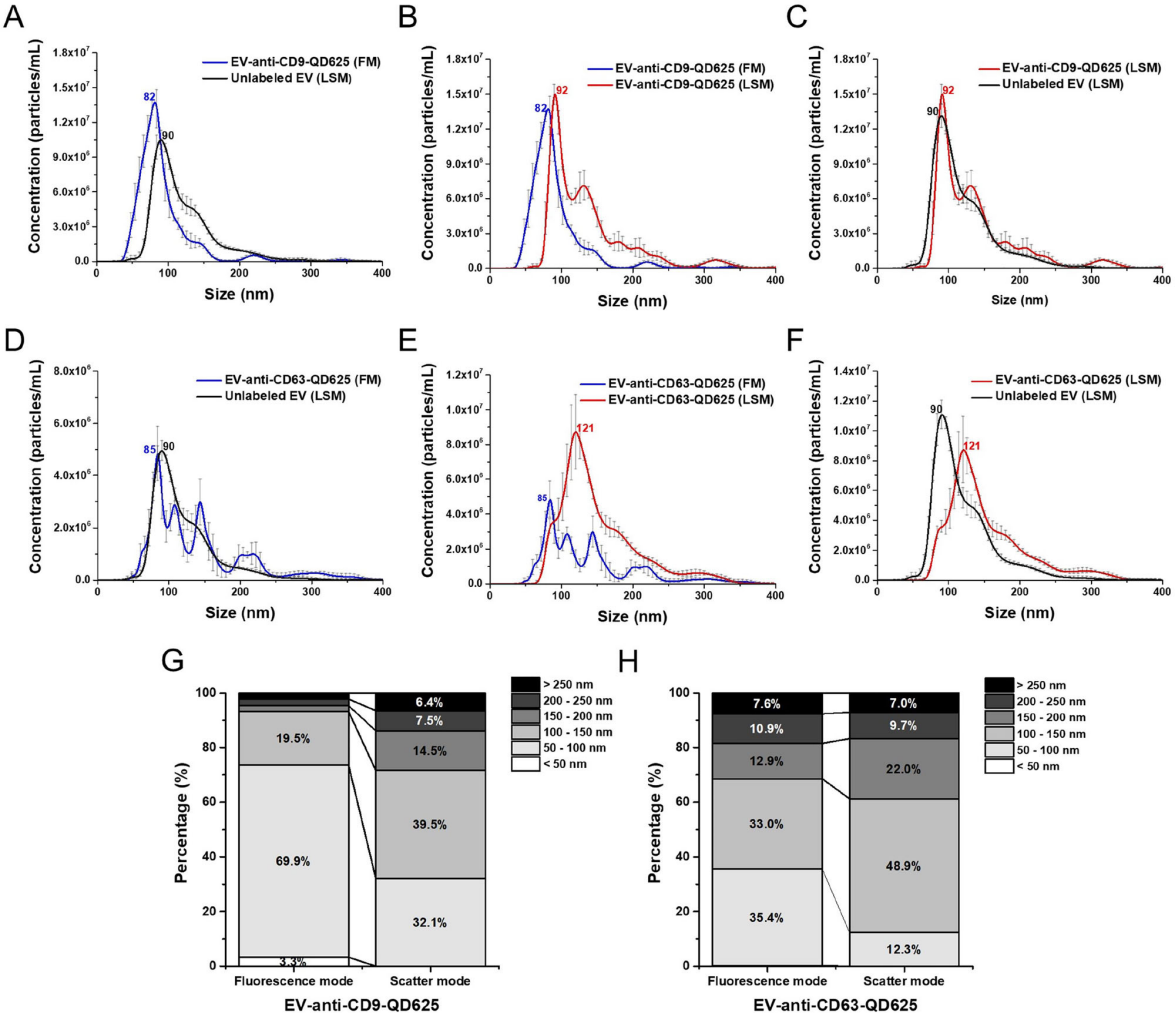
Comparative analysis of size distribution in scatter and fluorescence mode. The NTA plots show the comparison of size distribution of EVs under different conditions: unlabelled EVs measured in light scatter mode (black line), labelled EVs measured in fluorescence mode (blue line) and labelled EVs measured in light scatter mode (red line). Panels (A–C) represent data for CD9‐QD625, while panels (D–F) represent data for CD63‐QD625. All error bars represent SE (*n* = 3). (G, H) Stacked bar plots illustrate the percentage distribution of EV sizes in 50 nm intervals for both fluorescence and scatter modes, emphasising differences in EV subpopulations across the size ranges.

In the first comparison, we measured unlabelled EVs in LSM and labelled EVs in FM. The results revealed that FM detected particles in smaller size ranges compared to LSM (**Figure** **5A** **,D**). According to ISO 19430 standards, the lower limit of detection for Sc‐NTA is about 65 nm for biological materials (International Organization for Standardization 2016). However, QD labelling enabled the detection of particles as small as ∼30 nm, which is known to be the smallest size EVs can possess (Morales‐Kastresana et al. 2017). This improved size sensitivity allows for a more comprehensive analysis of the EV population.

The second comparison focused on labelled EVs in LSM and FM, with the only difference being the use or absence of a fluorescence filter. In LSM, without using a fluorescence filter, signals related to fluorescence can still reach the detector. Based on this, it can be predicted that the size distributions in these two modes would be similar, while differences might be observed in particle concentration. As shown in **Figure** **5B** **,E**, however, it has been confirmed that the size distributions between the two modes are different, with FM detecting particles in smaller sizes. We interpreted these results from two perspectives. The first perspective is the use of fluorescence filters. The scatter light from larger particles might mask the fluorescent signals of smaller particles, but the use of a long pass filter helps mitigate this effect. The second perspective focuses on understanding which size range the CD9 and CD63 antibodies predominantly bind to. In other words, it is necessary to determine the size ranges in which CD9‐ and CD63‐positive EVs are predominantly distributed (Guan et al. 2019; Saludas et al. 2022). **Figure** **5G** **,H** shows the distributions of CD9‐ and CD63‐positive EVs across 50 nm size intervals. In the size range above 150 nm, a noticeable decrease in the proportion of particles was observed in FM compared to LSM. This confirms that most EVs tend to bind more frequently to small EVs below 150 nm. However, differences were observed between CD9‐ and CD63‐positive EVs. When comparing the 50–100 nm and 100–150 nm size ranges, CD9‐positive EVs showed proportions of 69.9% and 19.5%, respectively, while CD63‐positive EVs exhibited proportions of 35.4% and 33.0%, respectively. This indicates that CD9‐positive EVs are more predominantly distributed among smaller EVs than CD63‐positive EVs.

Lastly, we conducted a comparison to confirm the size increase caused by QD labelling. As shown in **Figure** **5C** **,F** and **Table** **1**, the size changes between unlabelled EVs and labelled EVs were examined in LSM. For unstained EVs, the mean size in scatter mode was 120.1 ± 0.8 nm. EVs labelled with anti‐CD9‐QD625 exhibited a mean size of 141.4 ± 1.1 nm, while those labelled with anti‐CD63‐QD625 showed a mean size of 156.0 ± 2.7 nm. These results clearly indicate an increase in mean sizes when EVs were labelled with QDs. Importantly, this observed size increase correlated to the size of the QDs, suggesting that the labelling process contributes directly to the measured size changes (Zhang et al. 2020; Dobhal et al. 2018). Alternatively, the particle distribution observed in fluorescence mode can be interpreted as originating from smaller particles.

While these findings support improved size sensitivity using QD‐based fluorescence labelling, it is important to note that quantitative validation of marker‐positive EV subpopulations—such as CD9‐positive EVs—was not performed in this study. Techniques such as ELISA or bead‐based immunocapture could provide stronger confirmation and are proposed as essential next steps for future work.

### 3.5 Identification of EVs Subpopulations From Various Cell Lines

So far, we have evaluated the improvement in sensitivity, in terms of number concentration and size, compared to conventional methods when A549 cell‐derived EVs were stained with anti‐CD9‐QD625 and anti‐CD63‐QD625, respectively. Subsequently, we applied this methodology to multiple cell lines to identify EV subpopulations across different cell lines. The cell lines used in this study include EA.hy926 cells, commonly utilised in 3D lung model cultures, as well as THP‐1 cells and A549 cells (Marescotti 2019).

**FIGURE 6:**
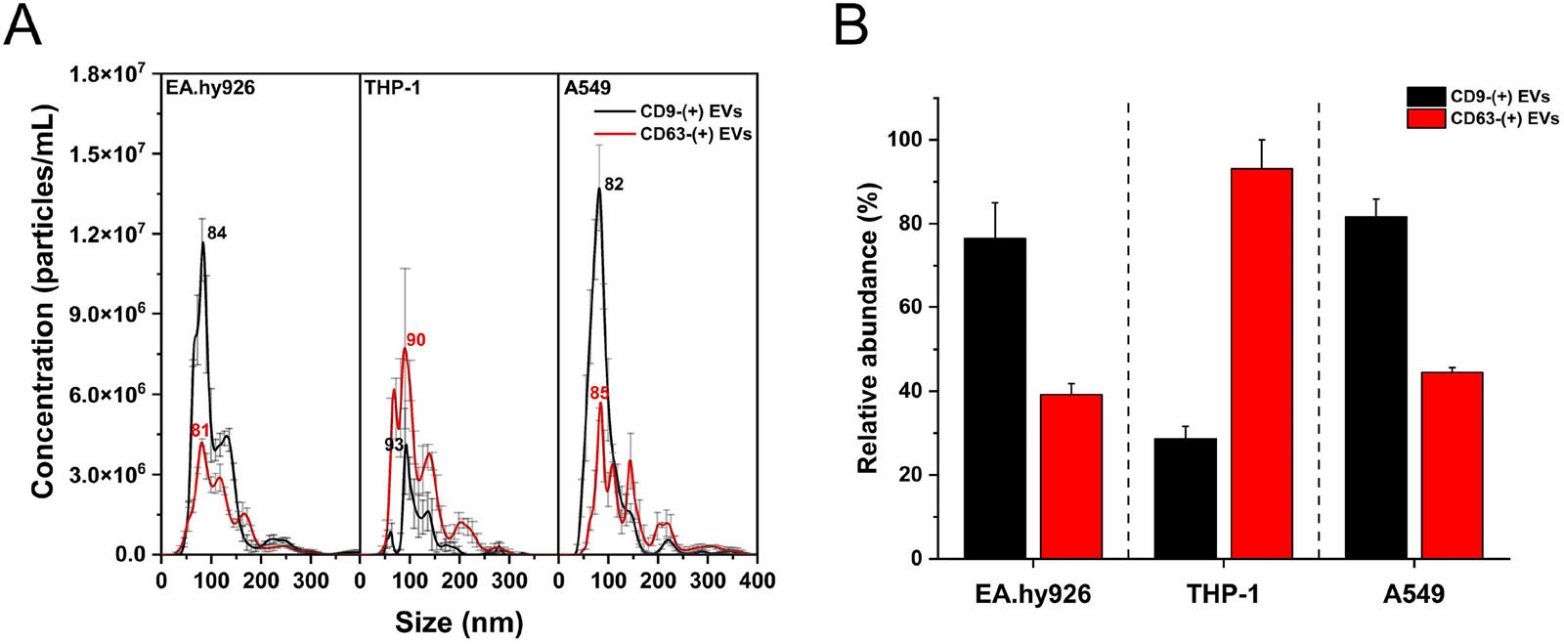
Identification of EVs subpopulations from various cell lines and their relative abundance. (A) Comparative size distribution profiles of CD9‐(+) (black line) and CD63‐(+) (red line) EV populations derived from EA.hy926, THP‐1 and A549 cells, measured in fluorescence mode. Error bars represent SE (*n* = 3). (B) Bar graph shows relative abundance of CD9‐positive and CD63‐positive EVs as a percentage, with error bars indicating SD (*n* = 3). Note that relative abundance was calculated as the number concentration ratio of FM to LSM.

**Figure** **6A** demonstrates the size distribution of CD9‐positive and CD63‐positive EVs from each cell line. Compared to the size distributions of unlabelled EVs (**Figure** **S3**), the results confirm that smaller EVs can be measured using Fl‐NTA than Sc‐NTA, consistent with the findings from A549‐derived EVs. Additionally, as shown in **Table** **2**, it was confirmed that CD9‐ and CD63‐positive EVs are more predominantly distributed among small EVs, a trend consistently observed in EVs derived from THP‐1 and EA.hy926 cell lines.

**TABLE 2:** Comparison of particle sizes of immunolabelled EVs derived from EA.hy926, THP‐1 and A549 cells.

NTA measurement mode	Size	EA.hy926 Cell‐derived EVs		THP‐1 Cell‐derived EVs		A549 Cell‐derived EVs	
		QD625 labelled					
		CD9‐(+)	CD63‐(+)	CD9‐(+)	CD63‐(+)	CD9‐(+)	CD63‐(+)
Scatter mode	Mean (nm)	125.6 ± 0.4	143.9 ± 1.3	125.3 ± 3.7	143.6 ± 4.2	141.4 ± 1.1	156.0 ± 2.7
	Mode (nm)	93.1 ± 6.7	102.7 ± 7.9	98.2 ± 6.5	102.8 ± 4.2	90.3 ± 1.4	118.1 ± 6.0
Fluorescence mode	Mean (nm)	110.0 ± 2.3	120.0 ± 2.9	92.0 ± 2.3	117.9 ± 2.5	95.7 ± 3.1	141.9 ± 2.7
	Mode (nm)	82.8 ± 3.7	82.7 ± 2.1	93.8 ± 7.3	85.0 ± 8.9	77.9 ± 3.1	103.8 ± 19.7

**Figure** **6B** illustrates the relative expression of CD9‐positive and CD63‐positive EV populations for each cell line. The results show that in EA.hy926 cells, similar to A549 cells, the CD9‐positive EV population is approximately 1.95 times more than the CD63‐positive EV population. In contrast, THP‐1 cells exhibit an inverse trend, where the CD63‐positive EV population is approximately 3.26 times larger than the CD9‐positive EV population. The observed differences in the CD9‐positive and CD63‐positive EV populations among the cell lines may be attributed to their distinct cellular origins and functions. EA.hy926 and A549 cells are lung‐derived endothelial and epithelial cells, respectively, while THP‐1 cells represent immune cells, specifically monocytes. These intrinsic differences in cellular characteristics likely contribute to the variations in EV marker expression. However, to confirm this hypothesis, further biological analyses are required to elucidate the underlying mechanisms driving these differences.

Despite the improvements introduced in this study, several limitations should be acknowledged. First, the use of PEG‐based precipitation, while effective for recovery, may co‐isolate protein aggregates and other non‐EV particles, which could influence immunolabelling and NTA analysis. Although rigorous washing steps and controls were employed, contamination cannot be entirely excluded. Second, the absence of CD81 labelling, a key tetraspanin marker commonly used in EV profiling, limits the comprehensiveness of EV subpopulation characterisation. This omission was due to the unavailability of validated QD–conjugated anti‐CD81 antibodies suitable for Fl‐NTA during the study period. Third, although Western blotting was used to confirm EV‐specific markers (CD9, CD63 and Hsp70), we did not assess the presence of potential cellular contaminants using negative markers such as calnexin. This limits our ability to fully validate the purity of the EV preparations and will be addressed in future studies. Fourth, no orthogonal techniques such as dSTORM or flow cytometry were used to independently validate EV marker expression or labelling specificity. Additionally, quantitative measures of sample purity, such as protein‐to‐particle ratios, were not included but could have provided a more rigorous assessment of EV isolation quality. Finally, the lack of standardised reference EV materials (e.g., CD9+/CD63+ bead standards) constrains the interpretability and reproducibility of the subpopulation quantification. These aspects will be addressed in future work to strengthen the robustness and generalizability of our findings.

## 4 Conclusion

In this study, we systematically optimised the conditions for EV immunolabelling using QDs and evaluated the advancements in detection sensitivity for Fl‐NTA with respect to particle concentration and size distribution. Our findings revealed that QD‐based labelling significantly enhanced the detection of EVs compared to conventional organic fluorescent dyes. Furthermore, this approach demonstrated the ability to detect smaller EV populations compared to Sc‐NTA analysis. Finally, this optimised methodology enabled the identification of a broad range of EV subpopulations across multiple cell lines, facilitating a comparative analysis of EV subpopulations unique to each cell type.

The improved EV characterisation achieved through Fl‐NTA combined with QDs‐based immunolabelling offers a precise and reproducible approach to effectively addressing the challenge of EV heterogeneity. By improving sensitivity through quantum dot‐based immunolabelling, this method establishes a robust framework for EV quality control, which is essential for both research and clinical applications. These advancements enhance the resolution of EV profiling and contribute to deeper insights into their biological functions and therapeutic potential.

## Author Contributions

**Eunyong Ha**: writing – original draft preparation, writing – review and editing, investigation, data curation, visualization. **Yewon Han**: writing – review and editing, investigation, validation. **Minseop Kim**: writing – review and editing, investigation, validation. **Zayakhuu Gerelkhuu**: writing—review and editing, validation**. Sook Jin Kwon**: validation. **Tae Hyun Yoon**: conceptualization, methodology, project administration, writing – review and editing, resources, supervision.

## Conflicts of Interest

The authors declare that they have no known competing financial interests or personal relationships that could have appeared to influence the work reported in this paper.

## Supporting information




**Supplementary Materials**: jex270072‐sup‐0001‐SuppMat.docx

## Data Availability

The data that support the findings of this study are available from the corresponding author upon reasonable request.
