# Supplementary material for: Quantum Dot‐Based Immunolabelling of Extracellular Vesicles and Detection Using Fluorescence‐Based Nanoparticle Tracking Analysis
Source: J Extracell Biol. 2025 Jul 22;4(7):e70072. doi: 10.1002/jex2.70072 (PMC12281464; doi:10.1002/jex2.70072)
Supplement: Supplementary file 1 — Supplementary Materials: jex270072‐sup‐0001‐SuppMat.docx [file JEX2-4-e70072-s001.docx]

**Quantum Dot-Based Immunolabeling of Extracellular Vesicles and Detection Using Fluorescence-Based Nanoparticle Tracking Analysis**

Eunyong Ha^a^, Yewon Han^a^, Minseop Kim^a^, Zayakhuu Gerelkhuu^b,c^, Sook Jin Kwon^c,d^, and Tae Hyun Yoon^a,b,c,d*^

^a^Department of Chemistry, College of Natural Sciences, Hanyang University, Seoul 04763, Republic of Korea.

^b^Department of Chemistry, Research Institute for Convergence of Basic Science, Hanyang University, Seoul 04763, Republic of Korea.

^c^Institute of Next Generation Material Design, Hanyang University, Seoul 04763, Republic of Korea.

^d^Yoon Idea Lab. Co. Ltd, Seoul 04763, Republic of Korea.

__________________________________________________________________________

*Corresponding authors. Tel: +82-(0)2-2220-4593

E-mail: [taeyoon@hanyang.ac.kr](mailto:taeyoon@hanyang.ac.kr)

# Supporting Information

Table S1. Comparison of concentration and immunolabeling efficiency of A549-derived EVs labeled with QD625 and Alexa488, measured by NTA in fluorescence and scatter modes.

**Figure S1.** Characterization of quantum dots (QD625). (A1-2) The size of QD625 was measured by TEM and (B) measured by NTA.

**Figure S2.** NTA instrument condition check using fluorescent polystyrene (PS) bead. The PS beads are tagged with Fluoresbrite Yellow Green. (A, B) represents the size distributions of 50 nm, 100 nm beads, respectively. The results confirm that the NTA accurately measures the bead sizes within their size CV values, which are 15% and 8% for the 50 nm and 100 nm PS beads, respectively.

Figure S3. Characterization of EA.hy926, THP-1 cell-derived EVs. The results show their majority size distribution, with the mode falling in the range of about 100 nm.

**Figure S4.** Quantification of EV-associated protein using the Bradford assay. (A) A standard calibration curve was generated using serial dilutions of bovine serum albumin (BSA), and absorbance was measured at 600 nm. (B) The protein concentration of EV samples was calculated by applying the measured absorbance value to the standard curve.

**Figure S5.** Size distribution profiles of A549-derived EVs under different incubation conditions as measured by NTA. (A) Immediately after isolation, (B) following 24-hour incubation in PBS, (C) following 24-hour incubation with PEG. Error bars represent SE (n=3).

**Figure S6.** NTA analysis of EV isolation medium containing exosome-depleted FBS.
Size distribution profile of media supplemented with 10% exosome-depleted FBS, as measured by NTA. Error bars represent SE (n=3).

# Tables

**Table S1.** Comparison of concentration and immunolabeling efficiency of A549-derived EVs labeled with QD625 and Alexa488, measured by NTA in fluorescence and scatter modes. Error bars represent SE (n=3).

| NTA measurement mode | Concentration | A549  cell derived EVs | | | |
| --- | --- | --- | --- | --- | --- |
|  |  | QD625 labeled | | Alexa488 labeled | |
|  |  | CD9-(+) | CD63-(+) | CD9-(+) | CD63-(+) |
| Scatter  mode | Number concentration  (particles/mL) | 8.27(±0.35) x 10^8^ | 6.98(±0.65) x 10^8^ | 5.59(±0.31) x 10^8^ | 7.07(±0.28) x 10^8^ |
| Fluorescence  mode | Number concentration  (particles/mL) | 6.61(±0.30) x 10^8^ | 3.12(±0.34) x 10^8^ | 6.42(±0.79) x 10^7*^ | 4.19(±0.15) x 10^7*^ |
| - | FM:LSM ratio | 79.9% | 44.5% | 11.5% | 5.9% |

^*^The valid track counts are below 200.

# Figures


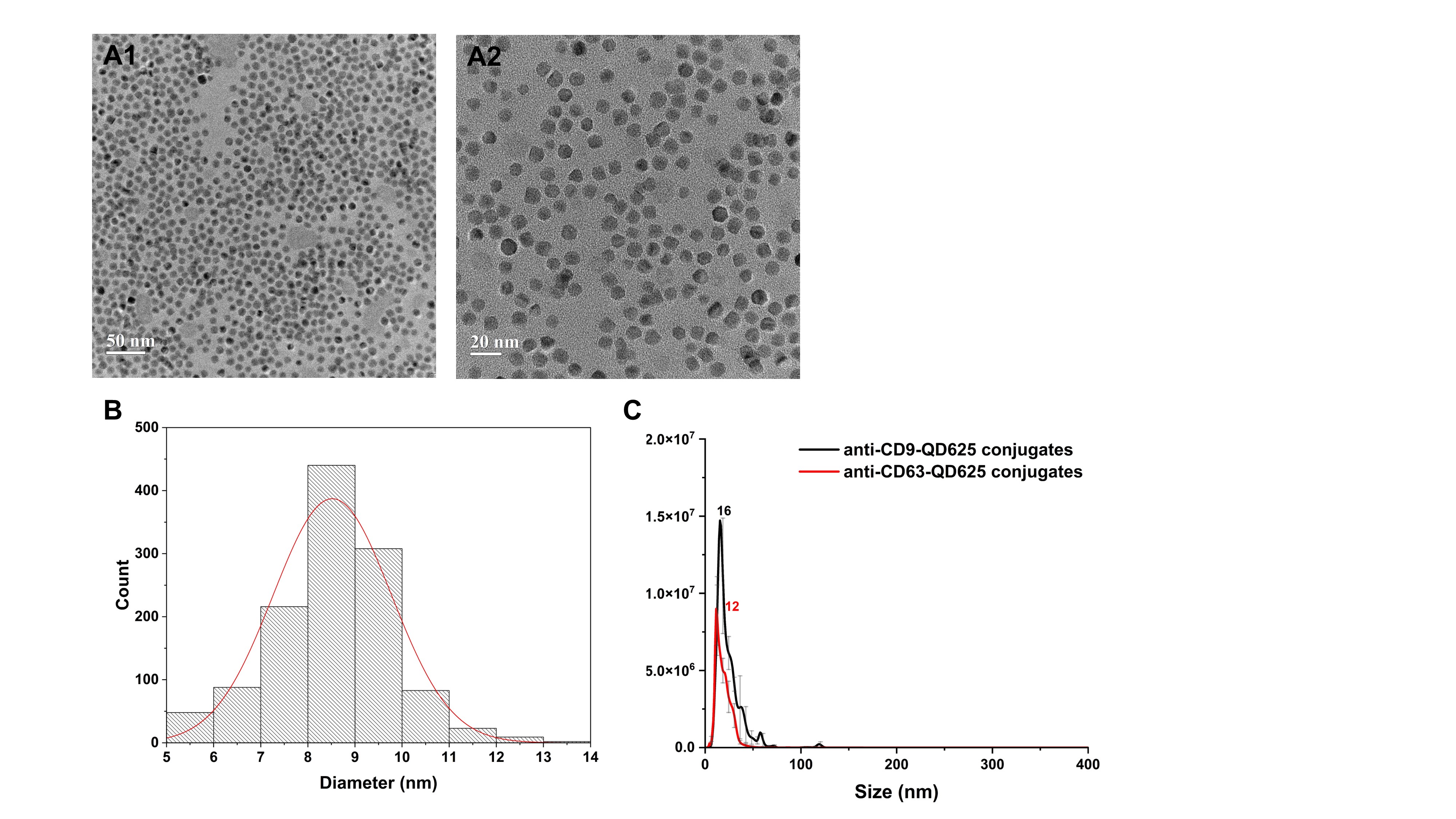


**Figure S1.** Characterization of quantum dots (QD625). (A1, A2) TEM images of QD625 at different magnifications. (B) Size distribution of QD625 measured from TEM. (C) Size distribution of anti-CD9-QD625 and anti-CD63-QD625 conjugates measured by NTA. Error bars represent SE (n=3).


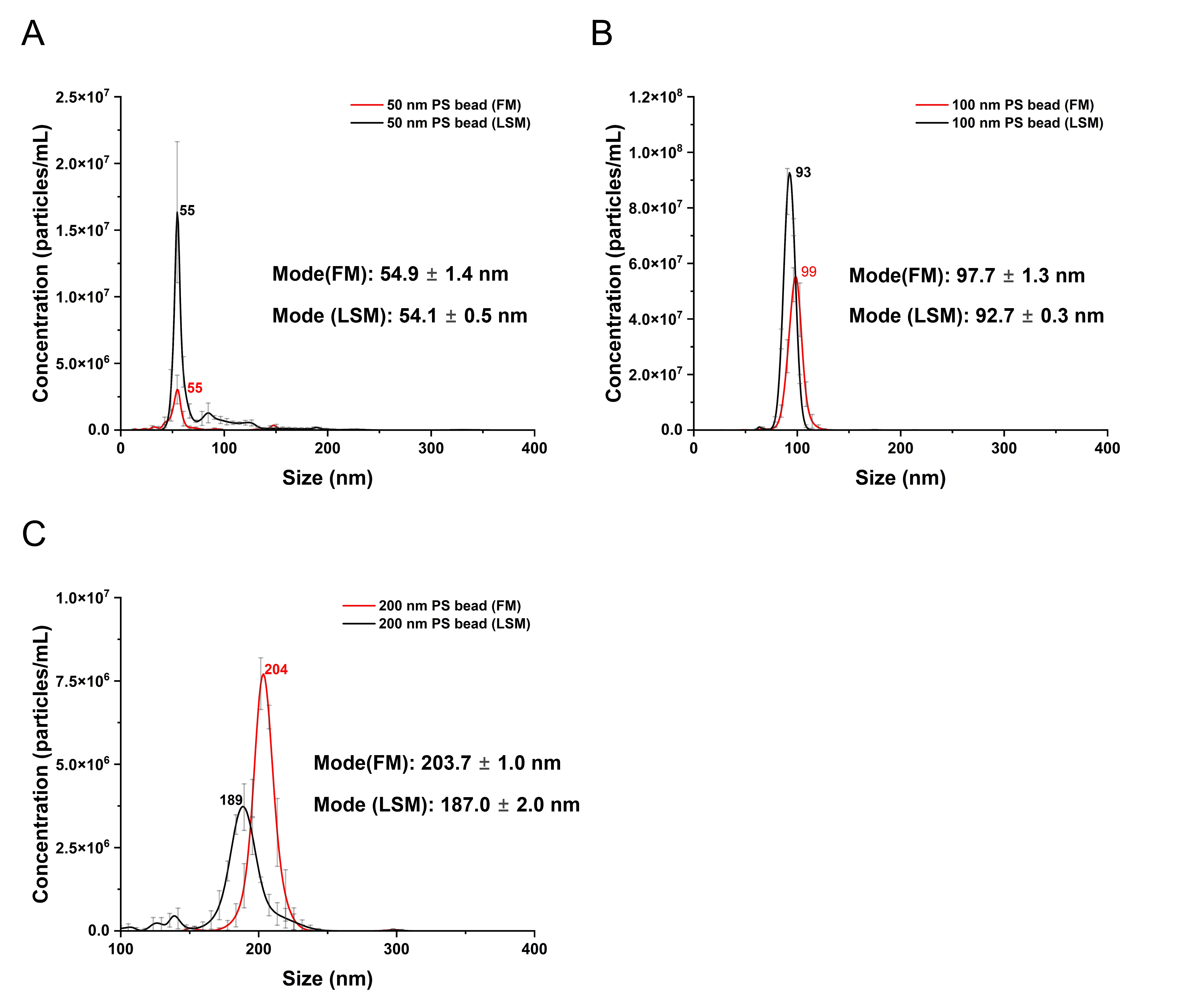


**Figure S2.** NTA instrument condition check using fluorescent polystyrene (PS) bead. The PS beads are tagged with Fluoresbrite Yellow Green. (A, B) represent the size distributions of 50 nm, 100 nm beads, respectively. The results confirm that the NTA accurately measures the bead sizes within their size CV values, which are 15% and 8% for the 50 nm and 100 nm PS beads, respectively. Error bars represent SE (n=3).


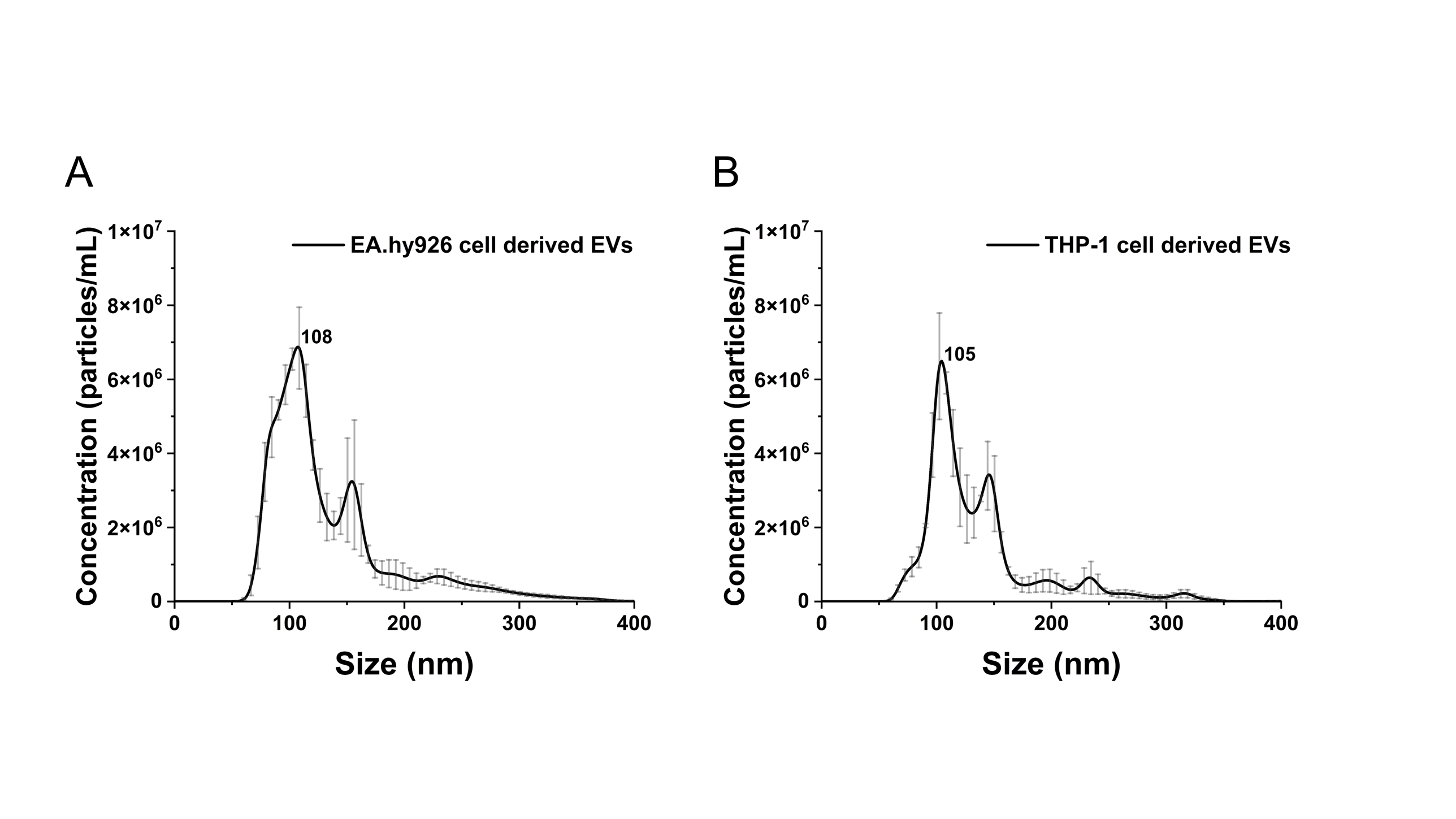


**Figure S3.** Characterization of EA.hy926 (A), THP-1 cell-derived EVs (B). The results shows their majority size distribution, with the mode falling in the range of about 100 nm. Error bars represent SE (n=3).


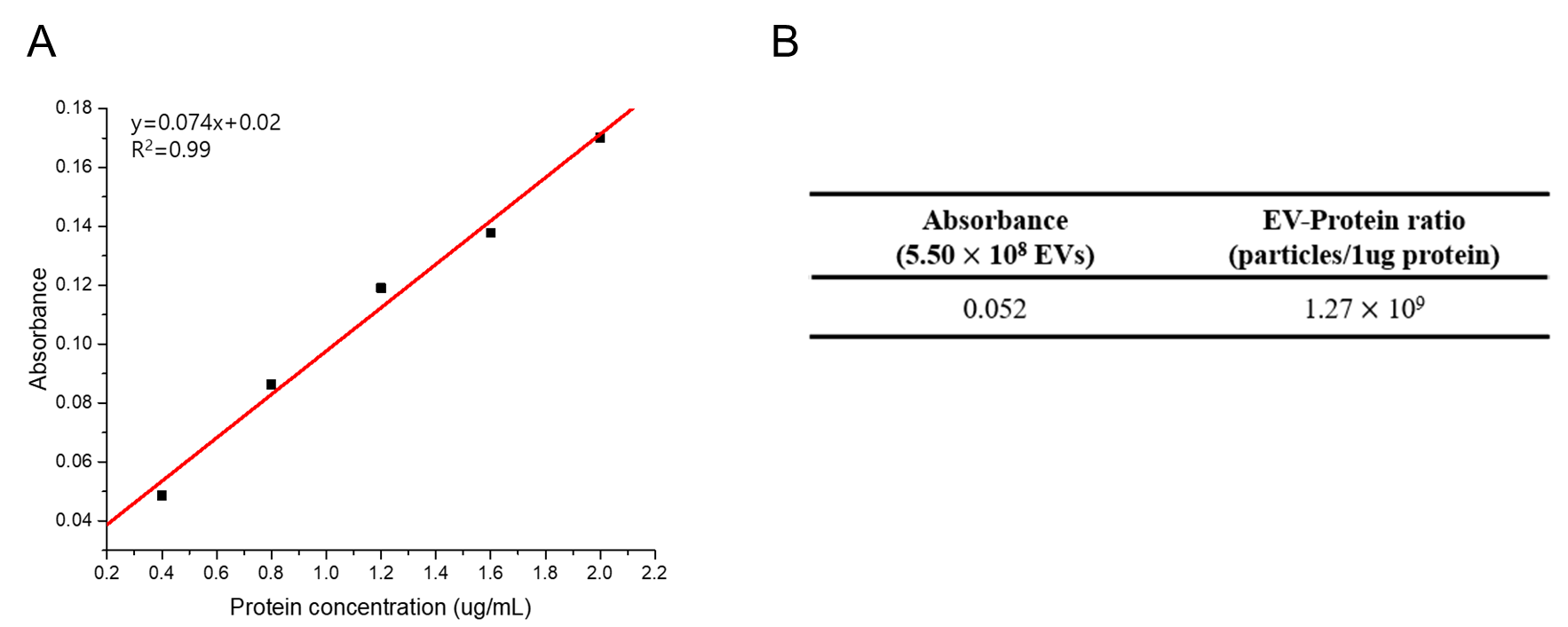


**Figure S4.** Quantification of EV-associated protein using the Bradford assay. (A) A standard calibration curve was generated using serial dilutions of bovine serum albumin (BSA), and absorbance was measured at 600 nm. (B) The protein concentration of EV samples was calculated by applying the measured absorbance value to the standard curve.





**Figure S5.** Size distribution profiles of A549-derived EVs under different incubation conditions as measured by NTA. (A) Immediately after isolation, (B) following 24-hour incubation in PBS, (C) following 24-hour incubation with PEG. Error bars represent SE (n=3).


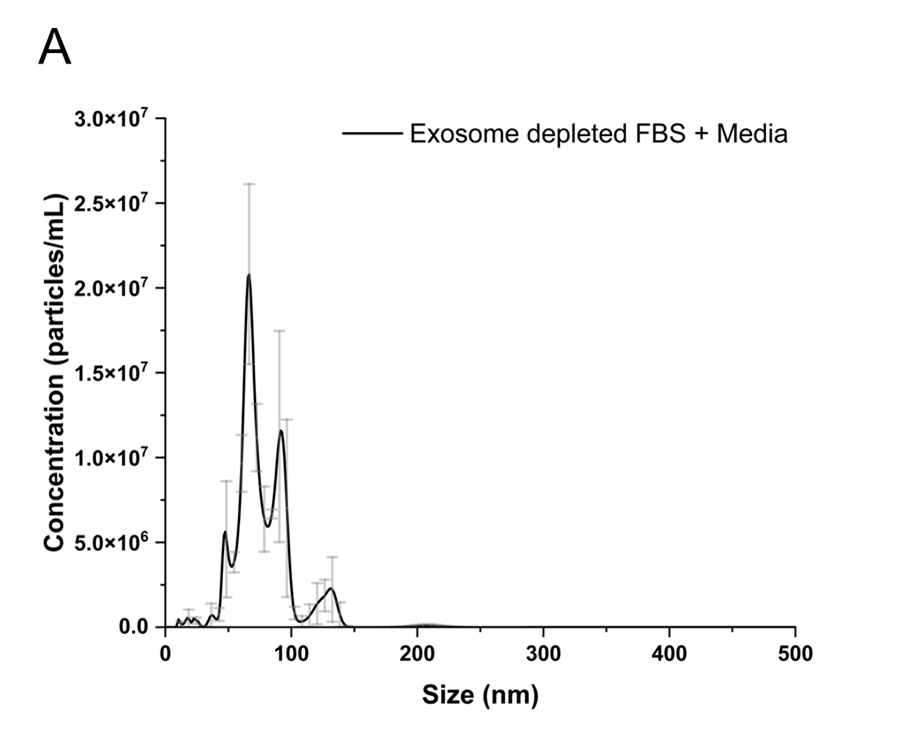


**Figure S6.** NTA analysis of EV isolation medium containing exosome-depleted FBS.
Size distribution profile of media supplemented with 10% exosome-depleted FBS, as measured by NTA. Error bars represent SE (n=3).

# List of figure captions (Supporting Information)

**Figure S1.** Characterization of quantum dots (QD625). (A1, A2) TEM images of QD625 at different magnifications. (B) Size distribution of QD625 measured from TEM. (C) Size distribution of anti-CD9-QD625 and anti-CD63-QD625 conjugates measured by NTA.

**Figure S2.** NTA instrument condition check using fluorescent polystyrene (PS) bead. The PS beads are tagged with Fluoresbrite Yellow Green. (A, B) represent the size distributions of 50 nm, 100 nm beads, respectively. The results confirm that the NTA accurately measures the bead sizes within their size CV values, which are 15% and 8% for the 50 nm and 100 nm PS beads, respectively.

**Figure S3.** Characterization of EA.hy926, THP-1 cell-derived EVs. The results shows their majority size distribution, with the mode falling in the range of about 100 nm.

**Figure S4.** Quantification of EV-associated protein using the Bradford assay. (A) A standard calibration curve was generated using serial dilutions of bovine serum albumin (BSA), and absorbance was measured at 600 nm. (B) The protein concentration of EV samples was calculated by applying the measured absorbance value to the standard curve.

**Figure S5.** Size distribution profiles of A549-derived EVs under different incubation conditions as measured by NTA. (A) Immediately after isolation, (B) following 24-hour incubation in PBS, (C) following 24-hour incubation with PEG. Error bars represent SE (n=3).

**Figure S6.** NTA analysis of EV isolation medium containing exosome-depleted FBS. Size distribution profile of media supplemented with 10% exosome-depleted FBS, as measured by NTA. Error bars represent SE (n=3).
